# Correction: EGFL7 loss correlates with increased VEGF-D expression, upregulating hippocampal adult neurogenesis and improving spatial learning and memory

**DOI:** 10.1007/s00018-023-04835-3

**Published:** 2023-07-13

**Authors:** Kathrin Barth, Verica Vasić, Brennan McDonald, Nora Heinig, Marc Christoph Wagner, Ulrike Schumann, Cora Röhlecke, Frank Bicker, Lana Schumann, Konstantin Radyushkin, Jan Baumgart, Stefan Tenzer, ·Frauke Zipp, Matthias Meinhardt, Kari Alitalo, Irmgard Tegeder, Mirko H. H. Schmidt

**Affiliations:** 1 Institute of Anatomy, Medical Faculty Carl Gustav Carus, Technische Universität Dresden School of Medicine, Fetscherstr. 74, 01307 Dresden, Germany; 2 Institute of Anatomy, University Medical Center of the Johannes Gutenberg University Mainz, Mainz, Germany; 3 Focus Program Translational Neuroscience (FTN), University Medical Center of the Johannes Gutenberg University Mainz, Mainz, Germany; 4 Institute of Medical Informatics and Biometry, Medical Faculty Carl Gustav Carus, Technische Universität Dresden, School of Medicine, Dresden, Germany; 5 Institute of Clinical Pharmacology, Goethe-University Hospital Frankfurt Am Main, Frankfurt, Germany; 6 Institute of Pathology, University Hospital Carl Gustav Carus, Technische Universität Dresden, Dresden, Germany; 7 Mouse Behavior Outcome Unit, Johannes Gutenberg University Mainz, Mainz, Germany; 8 Translational Animal Research Center (TARC), University Medical Center of the Johannes Gutenberg University Mainz, Mainz, Germany; 9 Institute of Immunology, University Medical Center of the Johannes Gutenberg University Mainz, Mainz, Germany; 10 Focus Program Immunotherapy (FZI), University Medical Center of the Johannes Gutenberg University Mainz, Mainz, Germany; 11 Department of Neurology, Rhine-Main Neuroscience Network (rmn2), University Medical Center of the Johannes Gutenberg University Mainz, Mainz, Germany; 12 Translational Cancer Medicine Program and iCAN Digital Precision Cancer Medicine Flagship, Faculty of Medicine, University of Helsinki, Helsinki, Finland

**Correction: Cellular and Molecular Life Sciences (2023) 80:54** 10.1007/s00018-023-04685-z

In this article the affiliation details for Authors processed incorrectly.

The correct affiliation should be as follow

Kathrin Barth^1^ ⋅ Verica Vasić^2,3^ ⋅ Brennan McDonald^1^ ⋅ Nora Heinig^1^ ⋅ Marc Christoph Wagner^1,4^ ⋅ Ulrike Schumann^1^ ⋅ Cora Röhlecke^1^ ⋅ Frank Bicker^2,3^ ⋅ Lana Schumann^6^ ⋅ Konstantin Radyushkin^3,7^ ⋅ Jan Baumgart^8^ ⋅ Stefan Tenzer^9,10^ ⋅ Frauke Zipp^3,10,11^ ⋅ Matthias Meinhardt^6^ ⋅ Kari Alitalo^12^ ⋅ Irmgard Tegeder^5^ ⋅ Mirko H. H. Schmidt^1^

^1^Institute of Anatomy, Medical Faculty Carl Gustav Carus, Technische Universität Dresden School of Medicine, Fetscherstr. 74, 01307 Dresden, Germany

^2^Institute of Anatomy, University Medical Center of the Johannes Gutenberg University Mainz, Mainz, Germany

^3^Focus Program Translational Neuroscience (FTN), University Medical Center of the Johannes Gutenberg University Mainz, Mainz, Germany

^4^Institute of Medical Informatics and Biometry, Medical Faculty Carl Gustav Carus, Technische Universität Dresden, School of Medicine, Dresden, Germany

^5^Institute of Clinical Pharmacology, Goethe-University Hospital Frankfurt Am Main, Frankfurt, Germany

^6^Institute of Pathology, University Hospital Carl Gustav Carus, Technische Universität Dresden, Dresden, Germany

^7^Mouse Behavior Outcome Unit, Johannes Gutenberg University Mainz, Mainz, Germany

^8^Translational Animal Research Center (TARC), University Medical Center of the Johannes Gutenberg University Mainz, Mainz, Germany

^9^Institute of Immunology, University Medical Center of the Johannes Gutenberg University Mainz, Mainz, Germany

^10^Focus Program Immunotherapy (FZI), University Medical Center of the Johannes Gutenberg University Mainz, Mainz, Germany

^11^Department of Neurology, Rhine-Main Neuroscience Network (rmn2), University Medical Center of the Johannes Gutenberg University Mainz, Mainz, Germany

^12^Translational Cancer Medicine Program and iCAN Digital Precision Cancer Medicine Flagship, Faculty of Medicine, University of Helsinki, Helsinki, Finland

The original article has been udpated.

